# Facile synthesis of efficient Co_3_O_4_ nanostructures using the milky sap of *Calotropis procera* for oxygen evolution reactions and supercapacitor applications

**DOI:** 10.1039/d3ra02555a

**Published:** 2023-06-16

**Authors:** Adeel Liaquat Bhatti, Aneela Tahira, Shusheel Kumar, Zaheer Ahmed Ujjan, Muhammad Ali Bhatti, Sooraj Kumar, Umair Aftab, Amal Karsy, Ayman Nafady, Antonia Infantes-Molina, Zafar Hussain Ibupoto

**Affiliations:** a Institute of Physics, University of Sindh Jamshoro 76080 Sindh Pakistan zaffar.ibhupoto@usindh.edu.pk; b Institute of Chemistry, University of Sindh Jamshoro 76080 Sindh Pakistan; c Institute of Chemistry, Shah Abdul Latif University Khairpur Mirs Sindh Pakistan; d Centre for Environmental Sciences, University of Sindh Jamshoro 76080 Sindh Pakistan; e Department of Metallurgy and Materials, Mehran University of Engineering and Technology 7680 Jamshoro Sindh Pakistan; f Department of Inorganic Chemistry, Crystallography and Mineralogy, Unidad Asociada al ICP-CSIC, Faculty of Sciences, University of Malaga, Campus de Teatinos 29071 Malaga Spain; g Chemistry Department, College of Science, King Saud University Riyadh 11451 Saudi Arabia; h Nanotechnology Research Centre (NTRC), The British University in Egypt (BUE) Cairo Egypt; i Department of Chemical Engineering, Mehran University of Engineering and Technology 7680 Jamshoro Sindh Pakistan

## Abstract

The preparation of Co_3_O_4_ nanostructures by a green method has been rapidly increasing owing to its promising aspects, such as facileness, atom economy, low cost, scale-up synthesis, environmental friendliness, and minimal use of hazardous chemicals. In this study, we report on the synthesis of Co_3_O_4_ nanostructures using the milky sap of *Calotropis procera* (CP) by a low-temperature aqueous chemical growth method. The milky sap of CP-mediated Co_3_O_4_ nanostructures were investigated for oxygen evolution reactions (OERs) and supercapacitor applications. The structure and shape characterizations were done by X-ray diffraction (XRD), scanning electron microscopy (SEM), energy-dispersive spectroscopy (EDS), and X-ray photoelectron spectroscopy (XPS) techniques. The prepared Co_3_O_4_ nanostructures showed a heterogeneous morphology consisting of nanoparticles and large micro clusters. A typical cubic phase and a spinel structure of Co_3_O_4_ nanostructures were also observed. The OER result was obtained at a low overpotential of 250 mV at 10 mA cm^−2^ and a low Tafel slope of 53 mV dec^−1^. In addition, the durability of 45 hours was also found at 20 mA cm^−2^. The newly prepared Co_3_O_4_ nanostructures using the milky sap of CP were also used to demonstrate a high specific capacitance of 700 F g^−1^ at a current density of 0.8 A g^−1^ and a power density of 30 W h kg^−1^. The enhanced electrochemical performance of Co_3_O_4_ nanostructures prepared using the milky sap of CP could be attributed to the surface oxygen vacancies, a relatively high amount of Co^2+^, the reduction in the optical band gap and the fast charge transfer rate. These surface, structural, and optical properties were induced by reducing, capping, and stabilizing agents from the milky sap of CP. The obtained results of OERs and supercapacitor applications strongly recommend the use of the milky sap of CP for the synthesis of diverse efficient nanostructured materials in a specific application, particularly in energy conversion and storage devices.

## Abstract 2

The preparation of Co_3_O_4_ nanostructures by a green method has been rapidly increasing owing to its promising aspects, such as facileness, atom economy, low cost, scale-up synthesis, environmental friendliness, and minimal use of hazardous chemicals.

## Graphical abstract

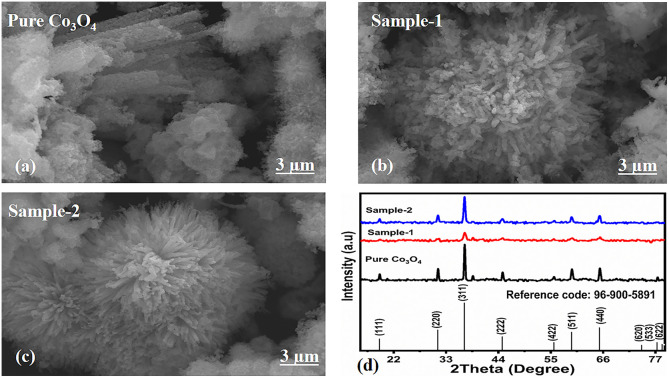

## 1. Introduction

**Scheme 1:**
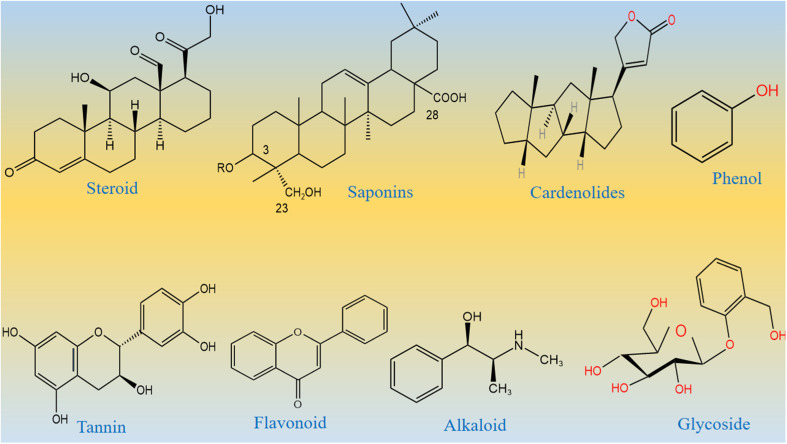
Molecular structures of major phytochemicals of the milky sap of CP.

From the Stone Age to nano era, energy is one of the most essential elements for the sustainability of life and environment. The use of energy relaxes and confronts the daily activities performed by us. Therefore, the demand for energy has increased rapidly over time due to the high population density of human beings and the development of a large number of industries.^1–5^ Most of the energy for our activities is provided entirely using fossil fuels, and therefore, fossil fuel deposits become smaller over time, and we cannot fulfill the requirement of energy from the fossil fuels. This critical scenario of increasing energy demands, depletion of fossil fuels, and their adverse impact on our environment necessitates the search for new alternative and renewable energy sources.^6–8^ Alternative energies are very sustainable and have been available since the beginning of the universe. However, there are certain barriers for the direct capitalization of available alternative energies. These alternative energies include solar, wind, water splitting, hydro power, and nuclear.^9–12^ Water splitting seems to be an emerging technology for lifting other technologies such as fuel cells and metal air batteries. Water splitting is a simple, efficient, and inexpensive method to strengthen renewable energy reservoirs.^13–17^ This proceeds *via* two well-known half-cell reactions, namely, hydrogen evolution reactions (HERs) and oxygen evolution reactions (OERs).^18–24^ From a thermodynamic point of view, the HER is simple as it uses two-electron transfer during the reaction, whereas the OER involves four-electron transfer during the reaction,^25–28^ and hence, the OER is very complicated and kinetically slow. Therefore, water splitting needs an active electrocatalyst for the realization of efficient HER and OER processes. To date, the state-of-the-art electrocatalysts for the HER are Pt-based materials and for the OER are Ru/Ir-based materials.^29–34^ These noble metal-based materials are scarce in nature and very expensive, and thus, they cannot be used for large-scale water splitting. Many efficient nonprecious electrocatalysts are reported for the HER, but we still need efficient non-noble electrocatalysts for the OER, since it is very difficult to transfer four electrons and form a double bond during the production of O_2_ molecules from water splitting. Research in this area is at its peak; however, the success for the practical production of O_2_ is still far from realization. Therefore, we must increase our efforts to develop efficient nonprecious catalysts from earth-abundant materials for the efficient OER. The transition metal oxides of iron, cobalt, nickel, and copper are active for the OER, but their performance is still inferior to that of the noble materials. Various electrocatalysts doped with cobalt oxide such as CoO_2_, Co (PO_3_)_4_, CuCo_3_O_4_, NiCo_3_O_4_, and MnCo_2_O_*x*_ are reported in the available literature.^35–42^ These studies show that these materials are neither stable nor durable under alkaline or acidic conditions. Several methods have been used to synthesize various metal oxides including solvent evaporation, electrochemical, hydrothermal, sol–gel, co-precipitation, and green-mediated approaches. The green production of metal oxides is among the synthetic methods with several advantageous features such as inexpensiveness, environmental friendliness, and simplicity. They are used very intensively in the present time.^43^ Large types of metal oxides have been synthesized by a green-mediated approach such as ZnO, Fe_2_O_3_, AgO, CuO, and Al_2_O_3_ using different plant extracts of *Agathosma betulina*, *Sida cordifolia*, *Pedalium murex*, *Gloriosa superba*, and *Prunus yedoensis*.^44^ The synthesis of different metal oxides such as ZnO, Fe_2_O_3_, AgO, CuO, and Al_2_O_3_ using various plant extracts has enhanced the functional properties of these materials towards specific application. This shows a strong motivation to adopt the use of new plant leaf extracts or fruit juice to improve the electrochemical properties of nanostructured materials, because the green-mediated approach offers a wide range of useful aspects in tuning the electrochemical performance of nanostructured materials such as the plentiful availability of reducing, capping, stabilizing and structure-orienting agents from various plant parts such as stems, leaves, flowers, fruits, and seeds.^45,46^ These beneficial phytochemicals from the plants prevent aggregation and control the shape and dimension of nanostructured materials.^45–47^ However, there is less attention paid to the use of green-mediated approach towards the synthesis of cobalt oxide (Co_3_O_4_) nanostructures and the role of green-mediated approach towards the enhancement in the electrochemical activity of Co_3_O_4_ nanostructures. Fortunately, Co_3_O_4_ is more stable and durable in alkaline media;^48–52^ however, its OER activity is low, hence new strategies and methods are required to accelerate the OER kinetics on the surface of Co_3_O_4_. In addition, Co_3_O_4_ is one of the transition metal oxides that have a high theoretical specific capacitance of about 3560 F g^−1^.^53–55^ The capacitance of Co_3_O_4_ in the practical applications is quite low compared to its theoretical value due to its restricted electron transfer, low electrical conductivity, limited surface area, contraction and large volume expansion, and aggregation of particles.^56–60^ These limitations of Co_3_O_4_ enabled the sluggish kinetics, poor capacitance and cycling stability during electrochemical testing. Therefore, the capacitance of Co_3_O_4_ needs to be increased by adapting new synthetic pathways. Among the abandoned plants, *Calotropis procera* (CP) is a species of flowering plant belonging to the family Apocynaceae that is native to North Africa, Pakistan, tropical Africa, Western Asia, South Asia, and Indochina. The main phytochemical components of CP include saponin, tannin, alkaloids, oxalate, phytate and cyanogenic glycosides. Many of these phytochemicals from CP have properties like reducing, stabilizing, and chelating agents; hence, they have been successfully used to enhance the electrochemical performance of Co_3_O_4_ nanostructures. The molecular structures of major phytochemicals in the milky sap of CP are drawn as shown in Scheme 1.

The use of natural products of the milky sap of CP to change the morphologies and electrochemical properties of Co_3_O_4_ is not reported; hence, it was used in the current work to tune the surface and optical structure of Co_3_O_4._ The ingredients of the milky sap of CP have improved Co_3_O_4_ electrical conductivity, created structural defects, improved catalytic sites, enhanced cycling stability, and lowered the OER overpotential for water oxidation in alkaline media.

In this study, we used the milky sap of CP as a surface-modifying reagent for Co_3_O_4_ nanostructures during the low-temperature aqueous chemical growth method. The ingredients of the milky sap of CP have improved the surface area, electrical conductivity, and OER activity of Co_3_O_4._ The physical structure of Co_3_O_4_ has been studied using different characterization techniques such as SEM, XRD, and EDS. The nanostructured Co_3_O_4_ showed excellent OER activity, with a low overpotential of 250 mV at 10 mA cm^−2^, and a low Tafel slope of 53 mV dec^−1^. A low Tafel slope of Co_3_O_4_ indicates a fast OER kinetics for practical applications. Co_3_O_4_ turned out to be very stable for a period of 45 hours and showed a low charge transfer resistance. The use of natural products of the milky sap of CP could be of great importance for the synthesis of a wide range of energy storage and conversion materials.

## 2. Experimental method

### 2.1. Used chemical reagents

Chemical reagents such as cobalt chloride hexahydrate (CoCl_2_·6H_2_O), urea, and potassium hydroxide (KOH) were obtained from Sigma Aldrich Karachi Pakistan. The milk of the Sodom apple was collected from the mountains of Jamshoro, Sindh Pakistan. All the desired solutions were prepared in deionized water.

### 2.2. Synthesis of Co_3_O_4_ nanostructures using the milky sap of CP

**Scheme 2:**
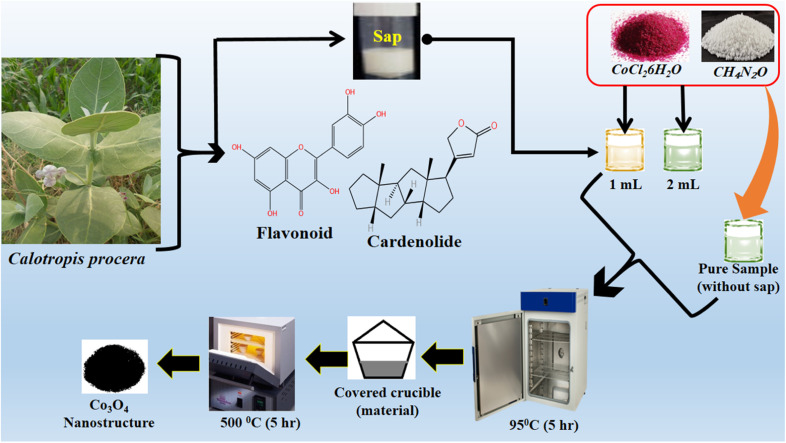
Stepwise preparation of Co_3_O_4_ nanostructures with the milky sap of CP.

In a typical synthesis, an equimolar (0.1 M) solution of cobalt chloride hexahydrate and urea solutions were prepared in 100 mL of deionized water in three separate 250 mL beakers. In two beakers, 1 mL and 2 mL of milky sap of CP were placed and they were labeled as sample 1 and sample 2 respectively. However, one beaker without the milky sap of CP was named pure sample. Then, these three beakers were sealed with aluminum foil and low-temperature aqueous chemical growth method was carried out in a preheated electric oven at 90 °C for 5 hours. Afterwards, the grown product was obtained on filter paper, washed with deionized water and dried overnight. Then, cobalt hydroxide was thermally decomposed at 500 °C in air for 5 hours, and finally, a black nanostructured product of Co_3_O_4_ was obtained. The synthesis process of Co_3_O_4_ is shown in Scheme 2.

### 2.3. Physical characterization of the prepared Co_3_O_4_ material using the milky sap of CP

The surface and crystal structures of the prepared Co_3_O_4_ material were investigated using a variety of analytical techniques such as XRD and SEM. The XRD experiment was performed with 1.5418 Å CuKα radiations at 45 kV and 45 mA. A SEM was used at an accelerating voltage of 3 kV, and an EDS equipped with a SEM was used to identify the chemical entities. The electrochemical characterization of the prepared Co_3_O_4_ materials was performed by cyclic voltammetry (CV) at 10 mV s^−1^, linear sweep voltammetry (LSV) at 5 mV s^−1^, and chronopotentiometry at 10 mA cm^−2^, and electrochemical impedance spectroscopy (EIS) was performed at 100 kHz to 1 Hz with an amplitude of 5 mV and OER onset potential in alkaline media. X-ray photoelectron spectroscopy (XPS) was performed for quantitative and chemical state information on the surface using an ESCA 5701 unit, Physical Electronics (PHI) working with monochromatic X-ray source Al (k-alpha) of photons at 1486 eV under ultra-high vacuum at a pressure of 10–10 mbar. The obtained XPS experimental results were analyzed using 0.651 eV Au 4f_7/2_ line of full width at half maximum. The UV-visible absorbance spectra were recorded using a UV-visible spectrophotometer (Lambda365, PerkinElmer, Waltham, MA, USA). The vibration bands of the Co_3_O_4_ material were obtained using a Fourier transform infrared (FTIR) instrument (Tensor 27, Bruker Optics, Karlsruhe, Germany).

### 2.4. Electrocatalysis and capacitance analysis on Co_3_O_4_ nanostructure materials

A three-electrode electrochemical cell assembly was fabricated using silver–silver chloride as the reference electrode, a platinum plate as the counter electrode, and a glassy carbon electrode (GCE) as the working electrode. The electrolyte was 1 M KOH de-aerated with N_2_. The ink of different Co_3_O_4_ materials was prepared by dispersing 5 mg in 2.5 mL of deionized water and 50 µL of 5% Nafion. The GCE was polished with 0.05µ alumina slurry and silicon paper. Then, 5 µL catalyst ink was coated onto the GCE by a drop-casting method. The modified GCE was ready for electrochemical measurements. CV was used first to confirm the stability of the material on the GCE, followed by LSV at slow scan to measure the OER activity. For measuring the electrochemically active surface area, CV was carried out at different scan rates. For monitoring the durability, chronopotentiometry was used at constant 10 mA cm^−2^. To understand the charge transport at the interface of the modified GCE and electrolyte, EIS was performed for the frequency range from 100 kHz to 1 Hz at an amplitude of 5 mV and OER onset potential as bias. All electrochemical tests were performed in 1.0 M KOH at room temperature. The measured silver–silver chloride potential is reported in reversible hydrogen electrode (RHE) using the Nernst equation. The capacitance experiments were conducted in 3.0 M KOH and the three-electrode cell set up.

## 3. Results and discussion

### 3.1. Physical studies of the morphology and crystalline aspects of Co_3_O_4_ nanostructures prepared with the milky sap of CP

**Fig. 1:**
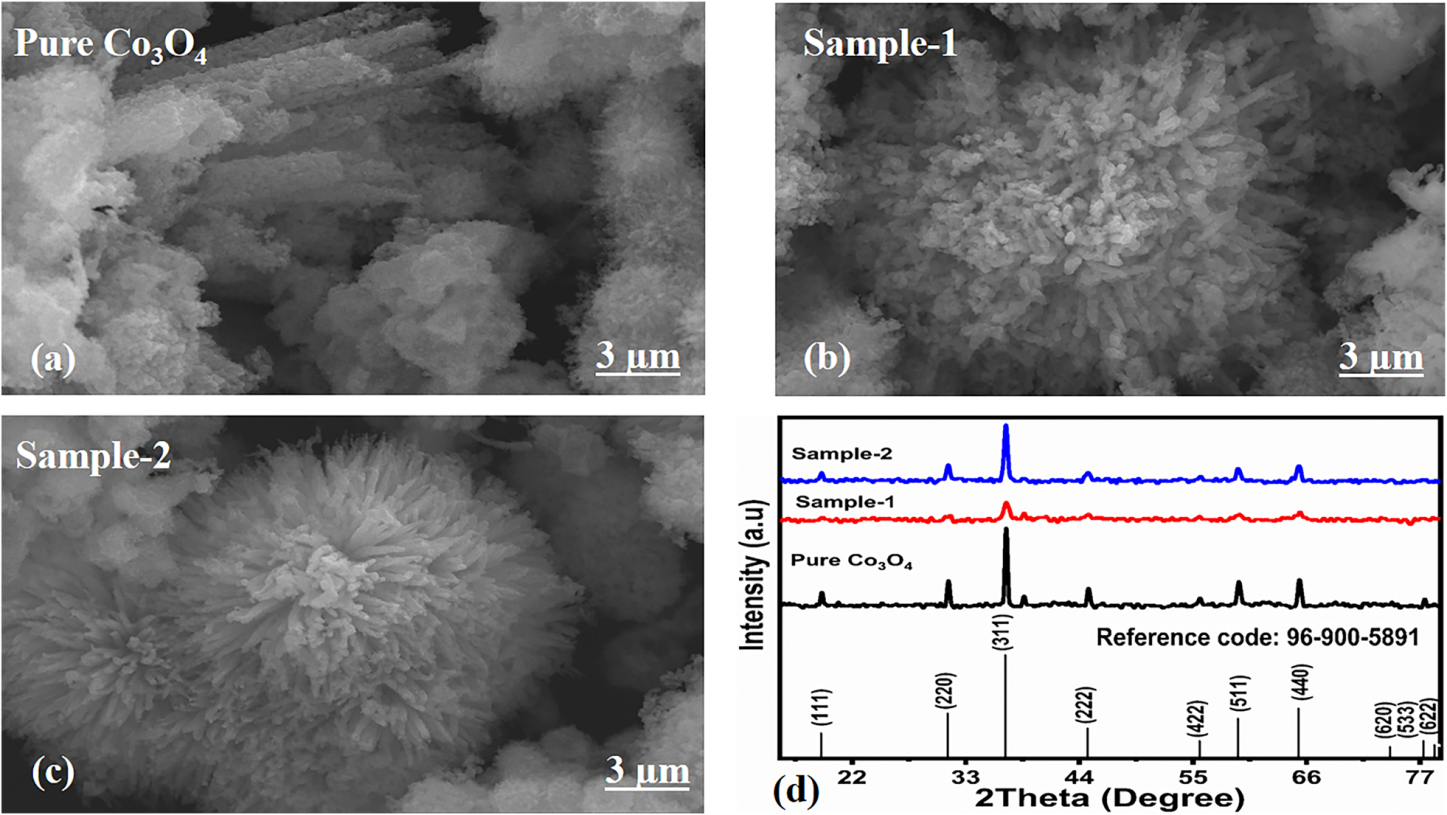
(a–c) Prominent SEM images of different samples including pure Co_3_O_4_, sample 1 and sample 2. (d) XRD reflection peaks of pure Co_3_O_4_, sample 1 and sample 2.

**Fig. 2:**
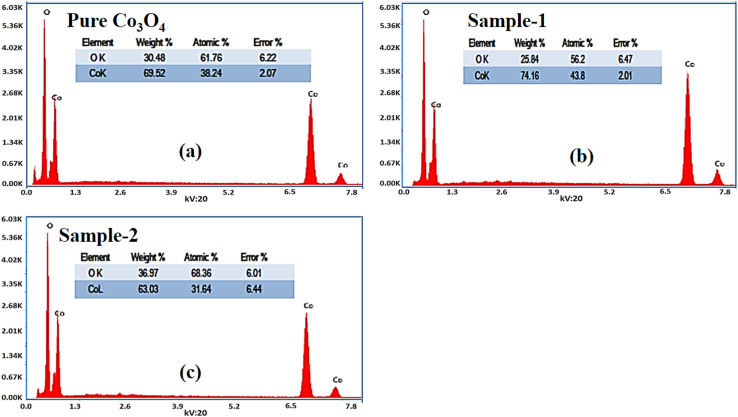
(a–c): Different EDS spectra including pure Co_3_O_4_, sample 1 and sample 2.

**Fig. 3:**
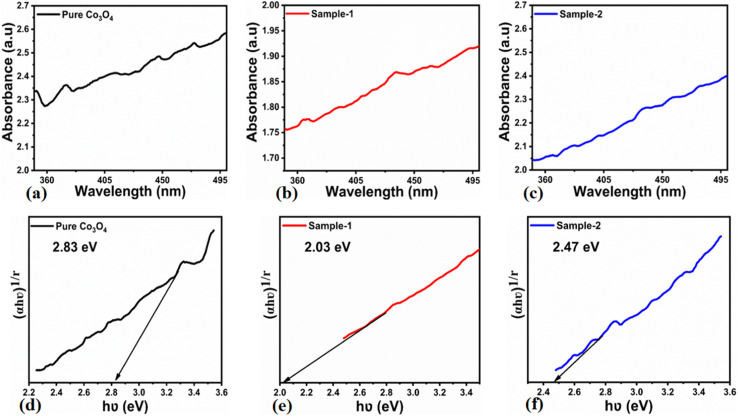
(a–c) UV-visible absorption spectra of pure Co_3_O_4_, sample 1 and sample 2. (d–f) Calculated optical band gap of pure Co_3_O_4_, sample 1 and sample 2.

The morphological studies of different Co_3_O_4_ nanostructures prepared with the milky sap of CP were performed using a SEM experimental tool, and the typical shape features recorded for pure Co_3_O_4,_ sample 1 and sample 2 are shown in Fig. 1a–c. It is evident that pure Co_3_O_4_ has nanorods covered by dense clusters that are several microns in size, indicating a high degree of heterogeneity of the pure sample. The morphology of Co_3_O_4_ made with different volumes of the milky sap of CP shows different shape orientations. The nanorods are lost and the clusters have been found for both the samples of Co_3_O_4_ prepared with 1 mL and 2 mL of CP, as shown in Fig. 1b and c. It is also evident that the size of the Co_3_O_4_ nanoparticles is smaller than that of the pure Co_3_O_4_ sample, confirming the role of CP's reducing, capping, and stabilizing agents in controlling the shape and size of nanostructured materials. From the SEM analysis, the use of phytochemicals from the milky sap of CP is highly favorable for enhancing the catalytic performance of nanostructured materials. The phase and purity of different Co_3_O_4_ nanostructures were studied by XRD measurement, and the recorded diffraction patterns are shown in Fig. 1d. The measured diffraction patterns were located at 2*θ* of 19.68°, 31.89°, 37.7°, 39.29°, 45.5°, 55.89° and 59.11°, assigned to the (111), (220), (311), (222), (400), (422) and (511) Miller indices respectively. All the diffraction patterns were attributed to the cubic phase of Co_3_O_4_ and well confirmed by standard JCPDS data (JCPDS 96-900-5891).^61,62^ It was seen that the relative intensities of the diffraction peaks (111), (222), (422), and (511) of the crystal planes were reduced in the case of sample 1, as shown in Fig. 1d. The use of the milky sap of CP did affect the crystalline properties of the crystal phase; however, there was no impurity in any sample and all the Co_3_O_4_ samples produced were of high purity. Furthermore, the Scherrer equation was used to estimate the values of the average crystallite size of different Co_3_O_4_ nanostructures, as given in Table 1. The measured values of the average crystallite size for pure Co_3_O_4,_ sample 1 and sample 2 were 26.05, 21.80 and 21.30 nm respectively. This analysis indicates that the milky sap of CP has shown negligible effects on the average crystallite size of sample 1 and sample 2, whereas pure Co_3_O_4_ possessed a higher crystallite size value than that of sample 1 and sample 2. From this information, it is obvious that the crystallite size did not play any role in the electrochemical performance of sample 1. Fig. 2a–c shows the typical elemental analysis of various Co_3_O_4_ nanostructure materials prepared with and without the milky sap of CP. The EDS spectrum of pure Co_3_O_4_ is enclosed in Fig. 2a. The Co_3_O_4_ nanostructures prepared with 1 mL and 2 mL of milky sap of CP are shown in Fig. 2b, c. The elemental analysis shows that Co and O were the major elements in each sample, and sample 1 contained a low amount of oxygen indicating the oxygen vacancies in sample 1. The atomic weight percentage of O in Co_3_O_4_, sample 1, and sample 2 was found in the order of 68.36%, 56.2% and 61.76% respectively, indicating the significant difference of sample 1 with respect to sample 2 and pure Co_3_O_4._ This low abundance of oxygen in sample 1 possibly would play an important role to accelerate the electrochemical activity. The atomic weight percentage of O sample 2 is still lower than that of pure Co_3_O_4_, however, it exhibits the atomic weight percentage of O slightly higher than that of sample 1. This has indicated the strong influence of different volumes of milky sap of CP on the variation in the oxygenated surface of Co_3_O_4._ The EDS study demonstrated the high purity of each sample. The optical band gap estimation was performed over synthesized Co_3_O_4_ with and without different amounts of the milky sap of CP using UV-visible measurements in the wavelength range of 200–700 nm, as shown in Fig. 3a–c. The absorbance values of Co_3_O_4_ with CP were higher than that of pure Co_3_O_4_, suggesting a strong indicator of the optical density of the prepared materials.^63^ The prepared Co_3_O_4_ materials with and without the milky sap of CP samples exhibit the absorbance edges in the visible part of each sample associated with multiple events of charge transfer between the metal and the ligand (O^2−^ → Co^2+^) and (O^2−^ → Co^3+^) of Co_3_O_4_.^64^ The milky sap of CP influenced the optical band gap variation of Co_3_O_4_ nanostructures and the Tauc formula shown below was used to quantify the optical band gap.^65^

1 (*αhν*)^*n*^ = *A*(*hν* − *E*_g_)

**Table 1:** Calculated average crystallite size of various Co_3_O_4_ nanostructures

Sample name	Peak position	FWHM	Crystalline size (nm)	Average crystalline size (nm)
Pure Co_3_O_4_	31.32	0.32333	25.51	26.05
	36.92	0.31482	26.60	
Sample-1	31.44	0.33	25.00	21.80
	36.9	0.45	18.61	
Sample-2	31.28	0.38	21.70	21.31
	36.88	0.4	20.93	

**Fig. 4:**
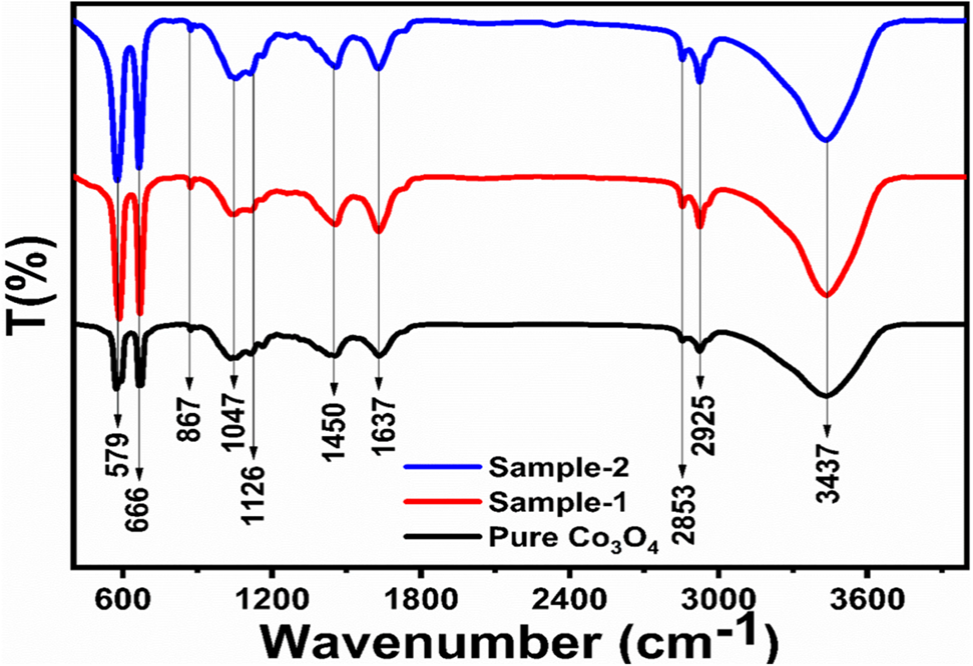
FTIR spectra of pure Co_3_O_4_, sample 1 and sample 2.

Different symbols in the above-mentioned equation refer to specific variable and constant values such as *A* as a constant, *α* is the absorption coefficient, *hν* is the photon energy and *E*_g,_ is the band gap energy and *n* is a constant value assigned to different types of transitions including 2, 1/2, 2/3 and 1/3 allowed direct, allowed indirect, forbidden direct and forbidden indirect.^66^ The simulated optical band gap of different Co_3_O_4_ nanostructures prepared with the milky sap of CP and pure Co_3_O_4_ using Tauc plots is shown in Fig. 3d–f. The estimated optical band gap of pure Co_3_O_4,_ sample 1, and sample 2 were in the order of 2.83 eV, 2.03 eV and 2.47 eV respectively, which again indicates the role of different volumes of the milky sap of CP on the optical features of Co_3_O_4._ Sample 1 was associated with a low optical band gap and would possess high conductance because Co_3_O_4_ is a semiconducting material and its electrical conductance is highly dependent on the optical band gap value. The variation in the volume of milky sap during the synthesis of Co_3_O_4_ has shown more pronounced surface plasmon bands, which could be associated with differences in the reduction characteristics, enhancement in the nucleation and size of crystals resulting from the oxygen vacancies, and surface defects, and hence, a difference in band gap calculation was observed. A FTIR study was conducted to evaluate the Co–O chemical binding properties of Co_3_O_4_ prepared with and without the milky sap of CP, as shown in Fig. 4. The FTIR spectra of all samples were recorded in the wave number range from 400 to 4000 cm^−1^. Several main vibrational bands were observed and corresponded to the presence of Co_3_O_4_ in each sample. The O–H stretching band positioned at 3437 cm^−1^ and 1637 cm^−1^ was assigned to absorb water molecules, as shown in Fig. 4. The nitrogen groups were also found at the band position of 1450 cm^−1^ and another connected 1126 cm^−1^ to the Co–OH coordinated bond. Typical metal–oxygen bands were noted at 450–650 cm^−1^. The FTIR study has described the presence of two bands associated with the octahedral and tetrahedral positions of Co^3+^ and Co^2+^ respectively, and this information has confirmed the formation of spinel structure Co_3_O_4_.^67^ The FTIR analysis was fully supported by previous studies.^68–70^

**Fig. 5:**
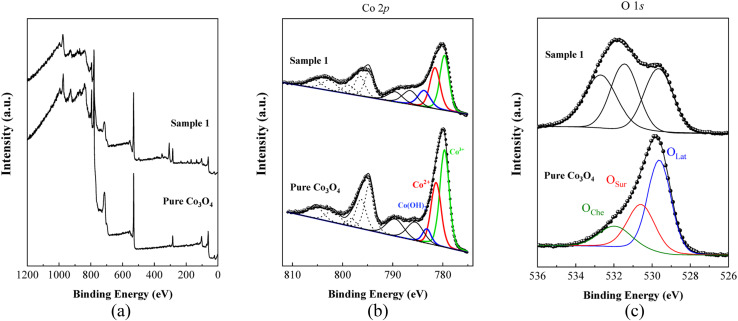
(a) Typical wide scan survey spectra of pure Co_3_O_4_ and sample 1. (b) Their resolved Co 2p_3/2_ spectra. (c) O 1s spectra of pure Co_3_O_4_ and sample 1.

The chemical composition and oxidation states of the different elements on the surface for the as-prepared pure Co_3_O_4_ and sample 1 were evaluated by XPS, and the corresponding signals are shown in Fig. 5. The wide scan survey spectra of pure Co_3_O_4_ and sample 1 are similar, where the presence of Co, O and C elements are evidenced in Fig. 5a. High-resolution spectra enabled the identification of the chemical states of cobalt and oxygen on the surface. In the case of Co 2p core level signals, the spectra were decomposed into three spin–orbit doublets (2p_3/2_–2p_1/2_) along with the corresponding shake-up satellites, consistent with a cobalt spinel structure and well confirmed by previous studies,^71,72^ as shown in Fig. 5b. By comparing the corresponding Co^3+^/Co^2+^ atomic ratios for pure Co_3_O_4_, 1.22, and A sample, 0.80, Co_3_O_4_ prepared with the milky sap of CP is richer in Co^2+^ at the surface. The high-resolution O 1s spectra for pure Co_3_O_4_ and sample 1 were also measured (Fig. 5c) and different types of oxygen environments such as lattice oxygen, surface oxygen, and chemisorbed oxygen were observed for both samples.^73^ This signal indicates that sample 1 shows major contribution from the oxygen surface (signal at *ca.* 531 eV), which is normally attributed to surface defects/vacancies. In this regard, the corresponding O_Sur_/O_Lat_ atomic ratio changed from 0.6 to 1.0 for pure Co_3_O_4_ and sample 1, respectively. Therefore, the presented data indicate that the milky sap of CP has induced the formation of a surface richer on Co^2+^ and oxygen vacancies, which could play an important role in the OER.

### 3.2. OER half-cell investigation of milky sap of CP-mediated sCo_3_O_4_ nanostructures

**Fig. 6:**
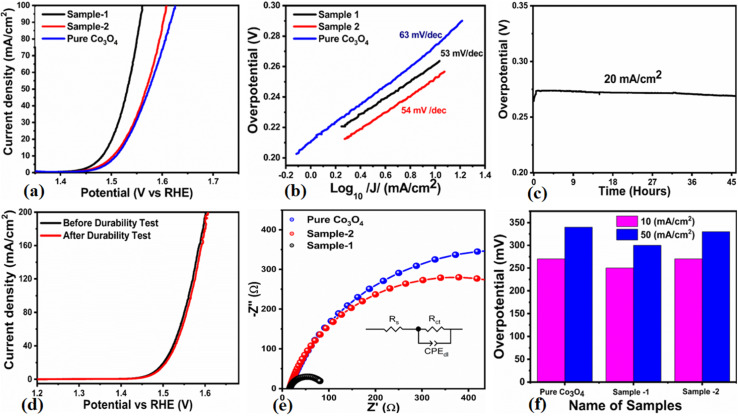
(a) Linear sweep voltammetry (LSV) polarization curves at a scan rate of 2 mV s^−1^ of pure Co_3_O_4_, sample 1 and sample 2 in 1.0 M aqueous KOH solution. (b) Tafel results. (c) Chronopotentiometry measurement about the durability of sample 1 at 20 mA cm^−2^ for 45 hours. (d) LSV before and after durability for the demonstration of stability. (e) Nyquist plots of pure Co_3_O_4_, sample 1 and sample 2 using electrochemical impedance spectroscopy (EIS) in the frequency range of 100 kHz to 0.1 Hz at an amplitude of 5 mV and onset potential of the OER; the inset shows an equivalent circuit. (f) Analysis of the overpotential at different current densities for pure Co_3_O_4,_ sample 1 and sample 2 with bar graph representation.

**Fig. 7:**
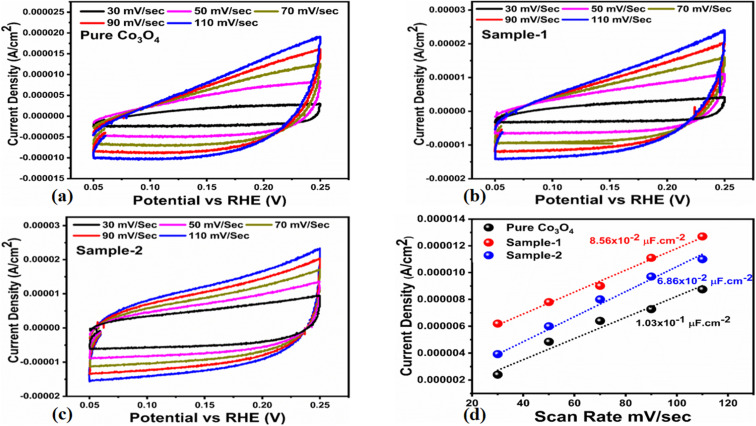
(a–c) Non-faradic CV scans at different scan rates for pure Co_3_O_4_, sample 1 and sample 2 in an electrolyte solution of 1.0 M KOH. (d) Calculated ECSA values of pure Co_3_O_4_, sample 1 and sample 2.

The OER activity for pure Co_3_O_4_, sample 1 and sample 2 was measured in a three-electrode cell setup with 1.0 M KOH by LSV at 5 mV s^−1^ (see Fig. 6a). The measured onset potentials for sample 1, sample 2 and pure Co_3_O_4_ are 1.45 V *versus* RHE, 1.48 V *versus* RHE and 1.51 V *versus* RHE respectively. The low OER onset potential for sample 1 suggests its excellent catalytic features, which could be attributed to the dynamic morphology, rich active sites and enhanced electrical conductivity using the Sodom apple as a surface modifier. The over potentials calculated at 10 mA cm^−2^ by subtracting the experimental RHE potentials from thermodynamic potential 1.23 V were found to be 250 mV, 270 mV and 280 mV for sample 1, sample 2 and pure Co_3_O_4._ Such calculations reveal that the use of natural products of the milky sap of CP as a surface modifier for Co_3_O_4_ has great potential to enhance the catalytic features of OER electrocatalysts by lowering the energy barrier for the OER process, which is the essential demand in the current studies, and they can also be used to design new functional materials for energy storage and conversion systems. To further understand the OER kinetics, the Tafel slopes were estimated from the linear region of LSV curves, as shown in Fig. 6b. The Tafel slopes obtained for sample 1, sample 2 and pure Co_3_O_4_ are 53 mV dec^−1^, 63 mV dec^−1^ and 66 mV dec^−1^ respectively. The low Tafel slope for sample 1 indicates the favorable and fast OER kinetics on its surface. Such a slow OER accelerated by the newly prepared Co_3_O_4_ material is a major advancement in the field of catalysis that could be used for practical applications. The Tafel slope of 53 mV dec^−1^ is the lowest to date for the Co_3_O_4_-based OER electrocatalysts in alkaline media. These results indicate the high potential of natural products for the design of efficient nonprecious catalytic materials. Chronopotentiometry was performed to monitor the durability of sample 1 at constant 10 mA cm^−2^ (see Fig. 6c). In addition, the milky sap of CP has significantly enhanced the durability of Co_3_O_4_-based sample 1 for 50 hours. There was no abruption in the potential during the test. This excellent durability of sample could be assigned to the good dispersion of Co_3_O_4_ and its fascinating structure. Based on this long-term durability experiment, sample 1 might be used for industrial applications. The stability of Co_3_O_4_-based sample 1 was evaluated by plotting the LSV curves before and after the durability test (see Fig. 6d). It can be observed that the OER onset potential was not altered even after the period of 50 hours, which reveals the outstanding stability of Co_3_O_4_-based sample 1. Hence, sample 1 has great potential for use in the production of O_2_ compared to the electrocatalysts under alkaline conditions recently reported for OERs. The EIS study was performed to gain a deep insight into the charge transport between the working electrode and the electrolyte, which favors the OER process, as shown in Fig. 6e. The EIS data were fitted with an equivalent circuit and the corresponding circuit elements were solution resistance (*R*_s_), charge transfer resistance (*R*_ct_) and constant phase element (CPE) corresponding to double-layer capacitance. The calculated charge transfer resistance values for pure Co_3_O_4,_ sample 1, and sample 2 were found as 823 ohms, 71 ohms, and 612 ohms respectively. The nanostructured Co_3_O_4_ sample prepared with low amounts of the milky sap of CP showed fast charge transport compared to pure Co_3_O_4_ and sample 2 of Co_3_O_4_ prepared with a large amount of the milky sap of CP, as shown in Fig. 6e. The small semi arc of the Nyquit plot is an indicator of low charge transfer resistance and the measured results agreed well with our previous study.^71^ Co_3_O_4_ is a semiconducting material and the optical band gap of sample 1 was calculated to be about 2.03 eV, which was lower than that of the pure Co_3_O_4_ sample and sample 2, hence the conductance of sample 1 is large, which is in good agreement with the EIS information about the charge transfer resistance experienced by sample 1 of 71 ohms. The combined results of optical band gap and EIS studies suggest that sample 1 exhibited high conductance, therefore it has shown highly favorable electrochemical performance. The performance evaluation of Co_3_O_4_ prepared with and without the milky sap of CP is presented graphically in terms of the estimated overpotential at two different current densities of 10 mA cm^−2^ and 50 mA cm^−2^, as shown in Fig. 6f. For better visualization, the overpotential of each material was estimated at two different current densities of 10 mA cm^−2^ and 50 mA cm^−2^, and is shown in Fig. 6f. The bar graph representation indicates that sample 1 exhibits a superior electrocatalytic activity even at a higher current density than that of pure Co_3_O_4_, and sample 2. To understand the reason for improved electrocatalytic activity of pure Co_3_O_4_, sample 1 and sample 2, we studied the electrochemically active surface area (ECSA) using CV curves in the non-redox region at different scan rates, as shown in Fig. 7a–c. There is a linear relationship between ECSA and the double-layer capacitance (*C*_dl_), hence we obtained the CV curves in the potential range of 0.05 to 0.25 *versus* Ag/AgCl (V) at different scan rates, as shown in Fig. 7a–c. The measured *C*_dl_ information of pure Co_3_O_4,_ sample 1, and sample 2 was estimated to be about 1.03 × 10^−2^ µF cm^−2^, 8.56 × 10^−2^ µF cm^−2^, and 6.86 × 10^−2^ µF cm^−2^ respectively, as shown in Fig. 7d. Sample 1 exhibited the highest value of ECSA, confirming the easy accessibility of active sites for the electrolyte, which clearly played an important role towards the enhanced OER. Furthermore, the OER performance was evaluated, and the recently published results of the OER are given in Table 2.^78–97^ It is obvious that the presented approach is facile, low cost, and efficient in terms of low overpotential, environmental friendliness and scale up for the fabrication of electrocatalyst materials.

**Table 2:** OER comparative analysis of sample 1 (Co_3_O_4_) with recently reported works

Catalyst	Overpotential @ 10 mA cm^−2^	Tafel slope (mV dec^−1^)	Electrolyte	References
CFAC/Ni oxide	370	66	1.0 M KOH	78
NiCo_2_S_4_	148	119	1.0 M KOH	79
FeCoO–NF	205	118	1.0 M KOH	80
NiCo_2_S_4_ hollow nanotube	330	95.7	1.0 M KOH	81
CO_3_O_4_/NICO_2_O_4_	294	97	1.0 M KOH	82
NiCo_2_O_4_–NC	300	156	1.0 M KOH	83
P-doped NiCo_2_O_4_ NWs/NF	290	88	1.0 M KOH	84
NiCo_2_O_4_ nanosheets	400	101	1.0 M KOH	85
NiCo_2_O_4_/NiMoO_4_ core/shell electrode	207	61	1.0 M KOH	86
NiCo_2_O_4_ nanosheet on Ni foam	385	96	1.0 M KOH	87
NiCo_2_O_4_/VN800	340	119	1.0 M KOH	88
CFP/NiCo_2_O_4_/Co_0·57_Ni_0.43_LMO	340	123	1.0 M KOH	89
CC@NiCo_2_O_4_	420	89	1.0 M KOH	90
NiCo_3_O_4_	420	51	1.0 M KOH	91
NiCoO_x_	300	80	1.0 M KOH	92
Ni–Co_3_O_4_	304	55	1.0 M KOH	93
NiCo_2_-2kcl	270	62	1.0 M KOH	94
Co_9_S_8_ NiCo_2_	290	74	1.0 M KOH	95
NiCo_2_O_4_ hollow microcuboids	213	49	1.0 M KOH	96
NiCo_2_O_4/_CU_*x*_O	317	84	1.0 M KOH	97
**Sample 1 (Co**_**3**_ **O**_**4**_ **)**	250	53	1.0 M KOH	**This work**

### 3.3. Capacitance analysis of milky sap of CP-mediated sCo_3_O_4_ nanostructures

**Fig. 8:**
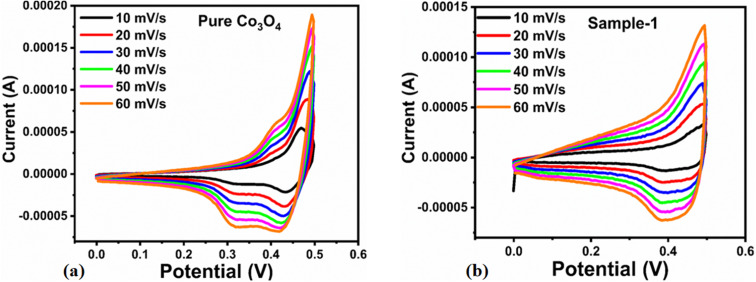
(a) CV scans of pure Co_3_O_4_.(b) CV scans of sample 1 at different scan rates in 3.0 M KOH to describe capacitance properties.

**Fig. 9:**
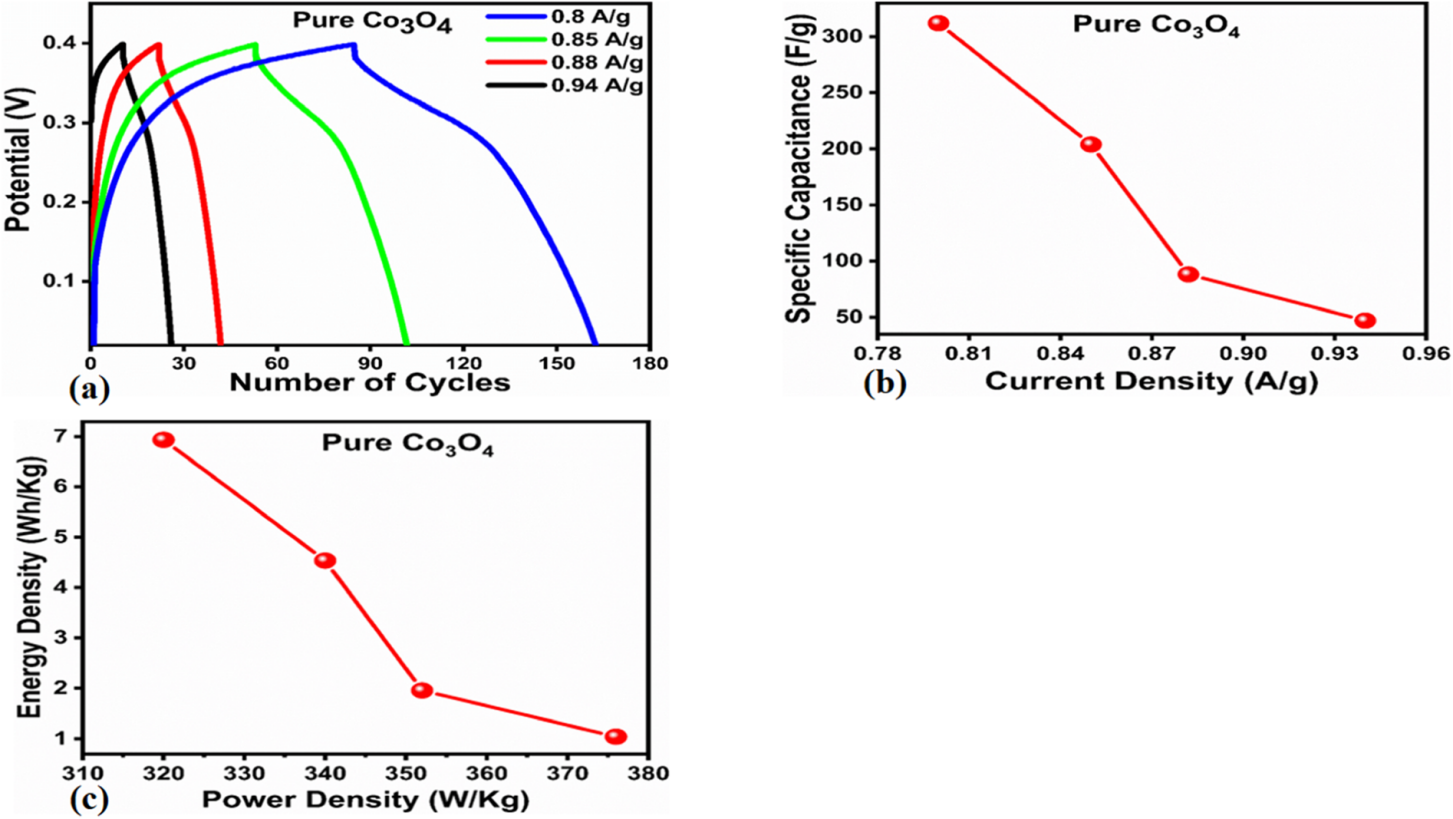
(a) GCD curves of pure Co_3_O_4_ at different current densities. (b) Specific capacitance. (c) Corresponding energy density and power density.

The capacitive activity of Co_3_O_4_ nanostructures prepared with the milky sap of CP was evaluated by CV using a three-electrode cell set up in 3.0 M KOH electrolyte. For comparison, pure Co_3_O_4_ was also used to evaluate the capacitance performance. The CV polarization curves were measured for milky sap of CP-mediated Co_3_O_4_ and pure Co_3_O_4_ in the potential window from 0.0 to 0.6 *versus* Ag/AgCl at different scan rates of 10, 20, 30, 40, 50 and 60 mV s^−1^, as shown in Fig. 8a,b. Both the milky sap of CP-mediated Co_3_O_4_ and pure Co_3_O_4_ nanostructures showed different shapes in the same potential window. Milky sap of CP-mediated Co_3_O_4_ and pure Co_3_O_4_ have shown a slight shift in the oxidation potential to a higher value, while the reduction potential towards a lower value with the increase in scan rate, as shown in Fig. 8a,b. This shift in oxidation and reduction of CV curves with the increase in sweep scan rates was assigned to the internal resistance and polarization effect.^74^ The nonlinear region of the CV curves confirms the redox properties of the milky sap of CP-mediated Co_3_O_4_ and pure Co_3_O_4_ and their pseudo capacitance properties. The pseudo-capacitance was found to be highly consistent with the increase in scan rate, suggesting an excellent reversibility of electrochemical redox processes.^75^ Moreover, the capacitance properties of pure Co_3_O_4_ were studied by employing galvanic charge–discharge (GCD) measurements at different current densities of 0.8, 0.85, 089, and 0.94 A g^−1^, as shown in Fig. 9a. The Ir drop in GCD curves as shown in Fig. 9a could be connected to the possible compressive stress, which increased the charging/discharging time, resulting in Ir drop. The GCD curves have shown the nonlinear behavior of pure Co_3_O_4_ and supported the claims about the pseudo-capacitance properties made on the CV analysis. The specific capacitance *C*_s_, energy density and power density were calculated using the following equations:^76,77^2
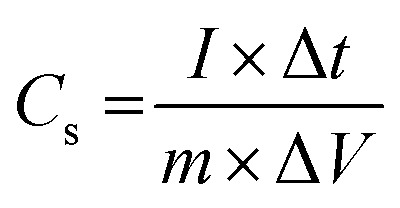
3
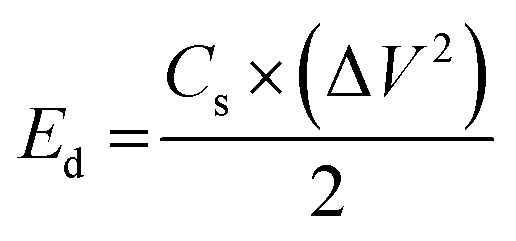
4
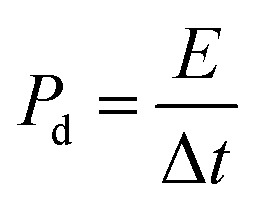


Herein, *C*_s_ is the specific capacitance, *I* is the current (A), Δ*t* is the discharge time (s), *m* is the deposited mass of material (mg), and Δ*V* is the potential window (V), *E*_d_ is the energy density, and *P*_d_ is the power density.

**Fig. 10:**
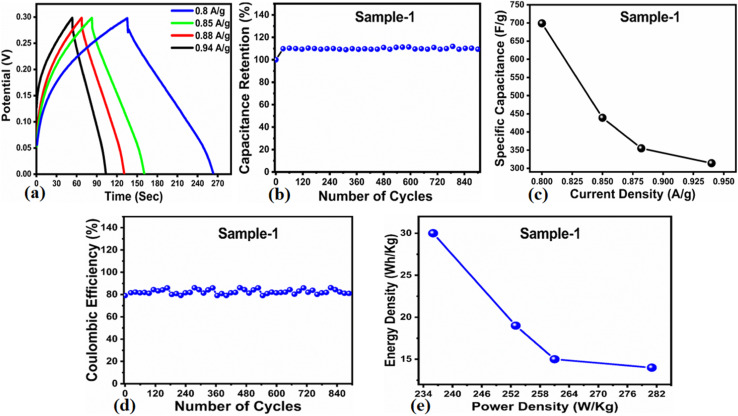
(a) GCD curves of sample 1 (Co_3_O_4_) at different current densities. (b) Percentage retention of specific capacitance. (c) Specific capacitance. (d) Percentage of coulombic efficiency. (e) Energy density and power density.

The *C*_s_ and energy density values of pure Co_3_O_4_ were estimated to be around 300 F g^−1^ at 0.8 A g^−1^ and 7 W h kg^−1^ respectively, as shown in Fig. 9b and c. This low value of specific capacitance indicates that there is immediate need to tune the capacitance properties of pure Co_3_O_4_, and hence, we prepared milky sap of CP-mediated Co_3_O_4_ with enhanced Cs and energy density, as shown in Fig. 10. The GCD curves of the milky sap of CP-mediated Co_3_O_4_ were measured at different current densities, as shown in Fig. 10a. It is worth noting that the nonlinear behavior is shown with almost a triangular shape and is fully supported by the CV redox behavior, which reveals an excellent pseudo capacitance behavior of milky sap of CP-mediated Co_3_O_4_. The specific capacitance retention percentage of 105% was observed for 900 cycles, which confirmed the excellent cycling stability of the material, whereas Cs of 699 F g^−1^ at 0.8 A g^−1^ was observed for milky sap of CP-mediated Co_3_O_4_, as shown in Fig. 10b and c. The retention rate of capacitance increased, as shown in Fig. 10b, after 900 GCD cycles due to the enhanced electron transport and high specific surface area exhibited by milky sap of CP-mediated Co_3_O_4._ This led to the generation of more charge transfer channels in the modified electrode, thereby accelerating the swift ion/electron transfer rate, which further improved the electrochemical activity of milky sap of CP-mediated Co_3_O_4_ and stabilized the working electrode.

**Scheme 3:**
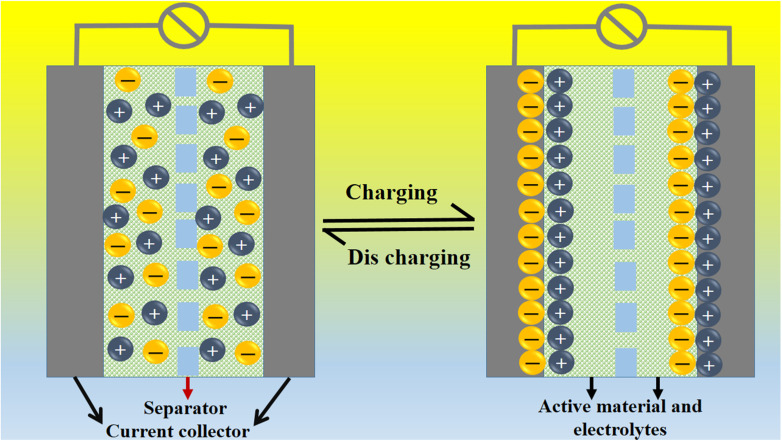
General energy storage mechanism.

A coulombic efficiency of more than 80% and an energy density of 30 W h kg^−1^ were estimated for milky sap of CP-mediated Co_3_O_4_, as shown in Fig. 10d and e. This significant progress in the enhanced capacitance properties of milky sap of CP-mediated Co_3_O_4_ could be attributed to the surface modification of nanostructures during the growth process in the presence of capping agents, reducing agents and stabilizing agents derived from the milky sap of CP. The enhanced electrochemical properties of milky sap of CP-mediated Co_3_O_4_ (sample 1) could be connected to the surface oxygen vacancies, low charge transfer resistance, low optical band gap, surface modification, and high ECSA values. The presence of capping agents, reducing agents and stabilizing agents together brought the surface modification and surface oxygen vacancies and enabled the high compatibility of milky sap of CP-mediated Co_3_O_4_ (sample 1), hence an enhanced electrochemical performance was demonstrated. The estimated values of specific capacitance, energy density, power density, coulombic efficiency, and specific capacitance retention percentages are given in Table 3 for the fast view of presented results. Moreover, we compared the presented capacitance results of milky sap of CP-mediated Co_3_O_4_ (sample 1) with many of the recent works, as given in Table 4,^98–102^ and hereby it is safe to say that the proposed material is better than or equal to many of the recently published supercapacitors in terms of specific capacitance and energy density. It has been shown that the working potential of Co_3_O_4_ was in the range of 0 to 0.45 V in alkaline media and the material exhibited pseudo capacitance features described by the CV curves shown in Fig. 8a and b. The mechanism of energy storage is shown in Scheme 3, which illustrates the typical charging-discharging behavior of the electro active material.^98,99^ The reversibility of valence state variation of Co^3+^/Co^4+^ confirms the quick redox properties of Co_3_O_4_ (sample 1). Additionally, the electrochemical redox potential of Co^3+^/Co^4+^ transition is shown to be almost the same, therefore the redox peaks are found to be closely overlapping.^100,101^

**Table 3:** Calculated Supercapacitor parameters of sample 1 (Co_3_O_4_)

Sample	Current density (A g^−1^)	Specific capacitance (F g^−1^)	Power density (W kg^−1^)	Energy density (W h kg^−1^)	Coulombic efficiency (%)	Capacitance retention (%)
Sample 1 (Co_3_O_4_)	0.8	699	236	30	86%	110%
	0.85	439	253	19		
	0.882	355	261	15		
	0.94	314	281	14		

**Table 4:** The compassion of sample 1 (Co_3_O_4_) capacitance results with some of the published works

Material	Specific capacitance	Current density	Potential window	Energy density (W h kg^−1^)	Power density (W kg^−1^)	References
CoNi–CNF	132 F g^−1^	1 A g^−1^	0–1 V	4.60	250	98
NCO@MWCNT	374 F g^−1^	2 A g^−1^	−0.5 to 2.2 V	95	3964	99
Co_3_O_4_@NiCo_2_O_4_ on carbon cloth\\AC	1198 F g^−1^	1 A g^−1^	1.5 V	30.6	133	100
MWCNTs	84 F g^−1^	0.6 A g^−1^	−0.5 to 2.2 V	21	6237	99
NCO//MWCNT	157 F g^−1^	0.6 A g^−1^	−0.5 to 2.2 V	40	2816	99
NCO@MWCNT//MWCNT	242 F g^−1^	0.6 A g^−1^	−0.5 to 2.2 V	61	2837	99
NiCoF	50.0	1 A g^−1^	0 to 1 V	—	—	101
NC6	1294.25	10 A g^−1^	0.4	—	—	102
**Sample 1 (Co**_**3**_ **O**_**4**_ **)**	699	0.8 A g^−1^	0 to 0.4 V	30	236	**This work**

## 4. Conclusions

In this study, we used the milky sap of CP to tune the surface properties of Co_3_O_4_ nanostructures by a low-temperature aqueous chemical growth method. Physical structure analysis showed a successful reduction in the optical band gap and shape of Co_3_O_4_ nanostructures by various reducing, capping and stabilizing agents. The newly prepared Co_3_O_4_ nanostructures proved to be highly active electrode materials for OERs and supercapacitor systems. We found the driving role of phytochemicals from the milky sap of CP in enhancing the electrochemical properties of Co_3_O_4_ nanostructures. The OER activity was supported by a low overpotential of 250 mV at 10 mA cm^−2^ and a long durability of 45 hours. The capacitance of Co_3_O_4_ nanostructures prepared with low amounts of the milky sap of CP was about 700 F g^−1^ at 0.8 A g^−1^ and the specific capacitance retention percentage was found to be about 105% after 900 GCD continuous cycles, revealing an excellent cycling stability of the material. The reducing, capping, and stabilizing agents from the milky sap of CP improved the morphology, particle size, and surface oxygen vacancies. The obtained results indicated that the synthesis of nanostructured materials using the milky sap of CP could be an effective method to fabricate high-performance electrocatalytic materials for the development of advanced electrochemical devices.

## Author contributions

Adeel Liaquat Bhatti, did the material synthesis and partial electrochemical tests. Aneela Tahira, did XRD analysis. Shushel Kumar, FTIR measurement. Zaheer Ahmed Ujjan, did supercapacitor tests. Muhammad Ali Bhatti, did the ECSA and optical band gap s measurements. Sooraj Kumar, did XRD measurement. Umair Aftab, did EIS analysis. Amal Karsy, did SEM measurement. Ayman Nafady, did preview of the results and validate them. Antonia Infantes-Molina, did XPS analysis. Zafar Hussain Ibupoto, did the supervision and wrote the original draft of manuscript.

## Conflicts of interest

Authors declare no competing interests in the resented research work.

## Supplementary Material
